# Adaptive multi-paddock grazing management’s influence on soil food web community structure for: increasing pasture forage production, soil organic carbon, and reducing soil respiration rates in southeastern USA ranches

**DOI:** 10.7717/peerj.13750

**Published:** 2022-07-19

**Authors:** David C. Johnson, Richard Teague, Steven Apfelbaum, Ry Thompson, Peter Byck

**Affiliations:** 1 Civil Engineering, College of Engineering, New Mexico State University, Las Cruces, NM, United States of America; 2 AgriLife Research, Texas A&M University, Vernon, TX, United States of America; 3 Department of Rangeland, Wildlife and Fisheries Management, Texas A&M University, College Station, TX, United States of America; 4 Applied Ecological Institute, Inc., Juda, WI, United States of America; 5 Resource Environmental Solutions, Broadhead, WI, United States of America; 6 School of Sustainability, Arizona State University, Tempe, AZ, United States of America; 7 Walter Cronkite School of Journalism, Arizona State University, Phoenix, AZ, United States of America

**Keywords:** AMP grazing, Soil food web, Carbon sequestration, Soil fertility & health

## Abstract

**Background:**

Measurement of two grazing management’s influence on pasture productivity, soil food web structure, soil organic carbon and soil microbial respiration efficiency was conducted on five southeastern US, across-the-fence ranch pairs to compare adaptive multi-paddock grazing (AMP) management, using short grazing events with planned, adaptive recovery periods, to conventional grazing (CG) management, with continuous grazing at low stock density.

**Methodology:**

A point-in-time experimental field analysis was conducted to compare five AMP or CG ranch pairs to better understand the influence of grazing management on (a) standing crop biomass productivity; (b) soil food web community population, structure and functionality; (c) soil organic carbon accrual; and d) soil-C (CO_2_) respiration kinetics.

**Results:**

AMP grazing systems outperformed CG systems by generating: (a) 92.68 g m^−2^ more standing crop biomass (SCB), promoting 46% higher pasture photosynthetic capacity (Two sample Mann-Whitney; Z = 6.1836; no DF in MW; *p* = 6.26 × 10^−10^; Effect size = 0.35) (b) a strong positive linear relationship of SCB with fungal biomass (R = 0.9915; F(1,3) = 175.35; *p* = 0.015); fungal to bacterial (F:B) biomass ratio (R = 0.9616; F(1,3) = 36.75; *p* = 0.009) and a soil food web proxy (R = 0.9616; F(1,3) = 36.75; *p* = 0.009) and a concurrent very strong inverse relationship with bacteria biomass (R = −0.946; F(1,3) = 25.56; *p* = 0.015); (c) significant predator/prey interactions with an inverse relationship with bacterial population biomass (*R* =  − 0.946; F(1,3) = 25.56; *p* = 0.015) and a positive relationship with total protozoa enumeration (R = 0.9826; F(1,3) = 83.68; *p* = 0.003) when compared to SCB; (d) a 19.52% reduction in soil C (CO_2_) respiration rates (Two sample *t*-test; T = −2.3581; DF = 52.3541; *p* = 0.0221; Effect size = 0.59); and (e) a 20.6% increase in soil organic carbon (SOC) in the top 10 cm of soil profile (Two sample Mann–Whitney; Z = 2.6507; no DF in MW; *p* = 0.008; Effect size = 0.24). Rancher conversion to AMP grazing strategies would appear to regenerate soil food web population, structure, diversity and biological functionality helping to improve: carbon flow into plant biomass, buildup of soil carbon, predator/prey nutrient cycling and soil microbial respiration efficiency while offering improved climate resilience and a strategy to increase the capture and storage of atmospheric CO_2_ in soils of the world’s rangeland.

## Introduction

This study reports on soil food web and vegetation interactions, under two grazing management strategies, an Adaptive Multipaddock (AMP) and a Continuous Grazing (CG) pasture management. It is part of a larger, multi-component southeastern USA study, focused on understanding the differences in the two management strategies. Research components and current published papers, and other components with publications pending, on this “systems” analysis are as follows: (1) soil carbon, nitrogen and water (Mosier et al., 2021), (2) greenhouse gas cycling (Gomez-Casanovas et al., 2021) (3) vegetation diversity and growth (Apfelbaum et al., 2022; Wang et al., 2021), (4) arthropod populations (Schmid, Welch & Lundgren, 2021), (5) grassland bird populations, (6) livestock well-being, (7) farmer well-being, (8) life-cycle cost assessment, (9) ecosystem modeling, and (10) production of documentary films https://www.carboncowboys.org/ (in progress) reporting on research results.

### Grazing Strategies

Grazing ecosystem soils and biotic communities coevolved with herbaceous vegetation, grazer insects and other herbivores over the last 40 million years, contributing to the expansion of carbon-rich soils in semiarid to semi-humid grassland regions that cover approximately 40% of the earth’s land area (Frank, McNaughton & Tracy, 1998; Retallack, 2013). The replacement of free-ranging wild herbivores, with fenced-in livestock, has contributed to degradation of vegetation and soils, declines in productivity and biodiversity, and a reduction in ecosystem resilience, resulting in an overall decline in historic ecosystem services generated through evolved grazer/grassland relationships (West, 1993; Milchunas & Lauenroth, 1993; Knopf, 1994; Frank, McNaughton & Tracy, 1998; Peterson, Allen & Holling, 1998).

To ensure sustainability and resilience, grazed ecosystems should be managed using grazing management that avoids overstocking and overgrazing (Teague, 2018). Conventional grazing management allows grazing animals unrestricted access in large fenced pastures, leading to degradation of soil and plant function due to patch-selective overgrazing and stocking with no planned recovery from grazing at stocking rates in excess of carrying capacity (Teague, 2018). Soil function is also diminished by the application of inorganic fertilizers, herbicides, pesticides, and parasiticides (Bardgett & McAlister, 1999; Leake et al., 2004; de Vries et al., 2006; Birkhofer et al., 2008; LaCanne & Lundgren, 2018). As an alternative, adaptive multi-paddock (AMP) grazing is a regenerative management option that has proven to be more productive, give greater economic returns and more abundant ecosystem services by improving biological function (Teague et al., 2013; Jakoby et al., 2014; Jakoby et al., 2015; Teague & Kreuter, 2020).

AMP emulates ecosystem processes that evolved in response to intense but periodic herbivory by large herds of ungulate grazers that included post grazing recovery (Frank, McNaughton & Tracy, 1998; Retallack, 2013) and has been documented to avoid and reverse the damage caused by continuous grazing in a timely and cost-effective manner (Gerrish, 2004; Teague et al., 2011; Teague et al., 2013; Wang, Teague & Park, 2016). AMP grazing uses a management decision process, responding to weather and forage variability, to avoid overstocking of, and overgrazing by livestock in an adaptive manner (Jakoby et al., 2014; Jakoby et al., 2015; Teague, Grant & Wang, 2015; Wang et al., 2018). The grazing benefits are achieved by adaptively adjusting grazing frequency and duration on available forage resources, using short, but intense grazing intervals, allowing livestock to graze ∼40% of available SCB. The remaining ∼60% of plant residue provides photosynthetic surface area to aid plant regrowth and/or be trampled onto the soil surface through short-term hoof-impact of the grazing herd. The trampled plant residue provides soil cover, reduces surface water runoff and erosion and provides above and below ground plant biomass to support microbial activity (Thurow, 1991; Dowhower et al., 2019; Teague & Kreuter, 2020). Appropriate length recovery periods promote more efficient recovery of plant communities, the soil food web and ecosystem restoration. Most AMP ranchers find this grazing strategy allows them to avoid application of inputs that may compromise soil biotic function, such as synthetic fertilizers, herbicides and pesticides. In combination, these actions result in light to moderate grazing impact on herbaceous plants, the soil food web, and the ecological functions they perform (Teague et al., 2013; Jakoby et al., 2014; Dowhower et al., 2019; Teague & Kreuter, 2020).

Mixed-study results from grazing researchers have concluded that no improvements in ecological and livestock indicators occurred under rotational grazing when compared to continuous grazing (*e.g.*, Briske et al., 2008). However, these conclusions have been criticized for their relatively long periods of grazing and short recovery periods, as well as the short-term and small-scale and fixed rather than adaptively managed research frameworks (Teague et al., 2013; Gosnell, Grimm & Goldstein, 2019; Gosnell, Charnley & Stanley, 2020; Teague & Kreuter, 2020). Other studies provide evidence that rotational grazing, with fewer paddocks per herd, more extended grazing periods, and shorter recovery periods, can result in limited plant and animal production advantages compared to continuous set-stocking at low stocking rates (Briske et al., 2008; Teague et al., 2013; Wang et al., 2022). Low stocking rates and improperly applied rotational grazing strategies do not facilitate degraded resource recovery or provide adequate economic returns over time (Jakoby et al., 2014; Jakoby et al., 2015; Teague & Kreuter, 2020). CG set-stocked at low stocking levels implementing long periods of grazing, paired with inadequate recovery, set-stocking rates, and low levels of soil surface forage residue promote a compounding of negative effects on ecological function and economic returns as illustrated by Augustine et al. (2020).

### Soil ecosystem

Intensification of agricultural production has promoted management practices that have reduced soil carbon and the biomass and diversity of soil microbiota (Stoate et al., 2001; Postma-Blaauw et al., 2010) including shifts to more bacterial-dominated microbial communities with increased soil nitrogen use (de Vries et al., 2006), and reduced soil carbon stocks and accrual rates (Six et al., 2006). Soil microbes are instrumental in C and N cycling; however, microbial biomass and activity are strongly influenced by higher trophic-level organisms of the soil food web. The upper trophic-level organisms (protozoa and nematodes) consume soil microbiota and stimulate microbial turnover through “grazing” of lower trophic level organisms (Postma-Blaauw et al., 2005) providing nutrient mineralization processes that promote plant productivity (Setälä & Huhta, 1991).

Soil organic matter (SOM), and the carbon and nutrients it contains, are key components for supporting fundamental bio-geochemical processes for: plant carbon assimilation and growth; soil respiration; and carbon-climate feedbacks (Kallenbach, Frey & Grandy, 2016). Soil food web population, structure, diversity, and biological functionality facilitate these bio-geochemical processes and contribute substantially to: nutrient generation, nutrient cycling, nutrient capture, soil fertility development, and SOM formation and turnover (Schloter, Dilly & Munch, 2003; Van der Heijden, Bardgett & Van Straalen, 2008; Murray et al., 2009; García-Orenes et al., 2013). A shift towards fungal dominance in the soil food web has been observed to enhance C accumulation and reduce SOM turnover rates (Six et al., 2006). More efficient microbial biomass production and the accumulation of SOM are now considered to be driven by distinct soil food web structures, where microbial-derived SOM is greatest in soils that contain higher fungal abundances (Kallenbach, Frey & Grandy, 2016; Johnson, 2017).

Predicting the effects of soil food web physiological regulation on soil C processes and their interaction with plants and livestock management, is critical if we are to improve the performance of our agroecosystems, project future global warming offset potentials, and enhance atmospheric CO_2_ reduction (Billings & Ballantyne, 2013). Despite this expectation, many studies have concluded there is no direct evidence that soil fungal-to-bacterial ratios (F:B) characterize the turnover of soil organic matter (Rousk & Frey, 2015), soil nutrient content or growth of vegetation (Wong et al., 2015), or that fungi are capable of enhancing soil carbon storage mechanisms (Thiet, Freya & Six, 2006; Johnson, 2017).

The influence of AMP grazing management on: *in-situ* field measurements for the soil food web population, structure, diversity and metabolic functionality, and the soil food web’s influence on standing crop biomass (SCB) production, soil organic carbon stocks (SOC) and soil microbial respiration efficiency is not well documented. The experimental hypothesis in this research is that AMP grazing methodologies will promote beneficial changes in soil food web population and structural composition (bacterial and fungal biomass, F:B ratio, and protozoa enumeration) and function, and that these shifts in the soil food web population structure will positively influence grazing system photosynthetic capacity, soil microbial respiration efficiency and the storage of carbon in AMP grazed rangeland soils when compared to CG systems. In this paper we evaluate the effects of two grazing management strategies on soil food web system functions and vegetation interactions measuring:

(a) early season standing crop biomass production (g dry biomass m^−2^);
(b) soil food web biomass (µg g^−1^ dry soil) for bacteria, actinobacteria, fungi; and population enumeration for total protozoa- (flagellates, ciliates, amoeba) (number g^−1^ dry soil);
(c) a fungal-to-bacterial (F:B) structural biomass ratio;
(d) a soil food web proxy (summation of all normalized soil food web constituents);
(e) soil organic carbon (SOC%); and,
(f) *in-situ*, diurnal soil CO_2_ respiration kinetics (g C m^−2^ day^−1^) of the soil food web,

in an effort to ascertain what can be learned about the soil food web’s contribution to the larger research team question: Can Adaptive Multi-Paddock (AMP) grazing contribute to sequestering carbon in soils and improve delivery of ecosystem services and socio-ecological resilience in grazing ecosystems?

## Materials & Methods

### Site screening, selection and soil type

A list of potential AMP and CG grazing partners was obtained from the Natural Resources Conservation Service (NRCS) technical staff, grazing consultants and organizers, in each of four states (Mississippi, Alabama, Tennessee and Kentucky) included in this study (Table 1). Potential candidates were invited to participate in an online survey to help in the selection of suitable paired ranches to conduct an across-the-fence comparison of adjacent AMP grazed and well-managed CG paired neighbors. Preliminary scouting was conducted on the prospective paired-ranches to confirm that each ranch pair had: (1) the same soil types, to reduce the potential that variable soil types may have on primary productivity, (2) similar topography, and (3) land use history with the primary deviation being the conversion of a former CG managed ranch to AMP grazing management that had been in AMP management for a minimum of one decade (Table 1, Table S1). Scouting included both soil typing using NRCS Soil Survey website (https://websoilsurvey.sc.egov.usda.gov/App/WebSoilSurvey.aspx) and pre-selection field soil core sampling and analysis to confirm accuracy.

**Table 1:** Description of ranch practices. Description of ranch practices; annual net primary productivity; livestock; paddock size, recovery and grazing periods; years of current management and land use history by ranch pair for adaptive multipaddock (AMP) and conventional grazing (CG).

Farm pair	Grazing practice	Total 2018 annual net primary productivity (g m^−2^)	Average animal units carried (AU ha^−1^)	Livestock in study area	Average paddock size (ha)	Average # of paddocks per herd	Graze period goal (days)	Recovery goal (days)	Rest vs. graze period (ratio)	Years of current management	Land use history
Statistical significance pooled (AMP vs. CG)		*p* = 0.3981	*p* = 0.011		*p* = 0.00025	*p* = 0.036	*p* = 0.0106	*p* = 0.9161	*p* = 0.01193	*p* = 0.3308	
Pair 1	AMP	1696	1.53	Beef, Sheep	1.2	45	2	45	22.5	13	Tobacco, grain, then grazing >30 years
	CG	1108	0.79	Beef Cattle	14	1	365	0	0	6	Tobacco and grain crops
Pair 2	AMP	892	2.57	Beef Cattle	1	45	2	90	45	12	Row Crops, Hay and Grazing
	CG	959	0.82	Beef Cattle	11	8	135	82.5	0.6	>25	Row Crops, Hay and Grazing
Pair 3	AMP	1119	1.55	Beef Cattle	1.2	60	1	50	50	29	Small Grains
	CG	1017	0.82	Beef Cattle	16	50	15	82.5	5.5	17	Small Grains
Pair 4	AMP	733	2.75	Beef Cattle	0.4	135	1	80	80	24	Cotton
	CG	496	0.97	Beef Cattle	18	2	365	0	0	>40	Cotton
Pair 5	AMP	924	1.04	Beef Cattle	1.6	150	1	70	70	10	Tobacco, grain the grazing >50 years
	CG	891	0.82	Beef Cattle	13	7	75	90	1.2	>40	Tobacco, cotton, market garden & grains

### Research area precipitation

Regional precipitation records, for the 6 months (January 2018–June 2018) prior to field sampling were obtained through NOAA’s Advanced Hydrological Prediction Service online data site (https://water.weather.gov/precip/). Heatmap images were obtained for the monthly percent-of-normal and April and May departure-from-normal precipitation maps for the Southeastern US corridor to confirm uniformity of precipitation events on research ranch pairs to verify similar growing conditions for assessing accurate SCB growth potential (Fig. S1).

### Soil organic carbon analyses

Soil sample cores for SOC analyses were collected as described in USDA/NRCS Soil Survey Laboratory Methods Manual (Burt, 2004) specifically, harvested in a three-week sampling period, between the last two weeks of May 2018 to the first week of June 2018. Ten catenas were located on the five AMP/CG ranch pairs. Twelve soil core samples were taken, at evenly spaced locations, across each of three pre-determined linear transects within each catena (sloped or flat), having rectangular dimensions of ∼100 m by ∼50 m (120 total soil core samples). The soil cores for SOC analyses were comprised of a soil core four cm in diameter and 10 cm deep taken at each sample site (12 sites at each AMP and CG farm), placed in 1-quart plastic bags and shipped to Ward Laboratory Inc., (4007 Cherry Ave, Kearney, NE 68847) for SOC (%) carbon analysis (LECO, dry combustion). Laboratory testing included a preliminary acidification treatment to remove soil carbonate content and provide an accurate SOC (%) assessment. Each of the sample cores were sampled in triplicate to insure SOC (%) sampling accuracy.

### Standing crop biomass (SCB)

Standing crop biomass was assessed (g dry biomass m^−2^) by clipping living/standing plant biomass 2.54 cm above ground height, assessing from 15–20 individual quadrats (quadrat = 1,000 cm^2^) taken inside multiple fenced livestock-exclusion areas, in each catena, on AMP and CG pastures. All plant materials were paper bagged, oven dried and weighed for dry biomass. The sample sites (sloped or flat) corresponded with the microbiological sampling sites for each ranch pair (Table S1) for the January to early June pasture forage growth period in each treatment plot. Each sample site was harvested, in a three-week sampling period, between the last two weeks of May 2018 to the first week of June 2018 corresponding with the sample timing for all microbiological and respiration metrics.

### Soil food web analyses

Soil samples for soil food web analyses were collected as recommended by Earthfort (https://earthfort.com/wp-content/uploads/2021/08/Sample-Instructions-Combined_81221.pdf), specifically sampling at each location from a soil core four cm in diameter and 10 cm deep of the soil profile (*n* = 12 for each ranch). These samples were immediately placed on ice and mailed overnight to Environment Celebration Institute Inc., (Berry Creek, CA, USA) for soil food web analyses to quantify soil food web biomass (µg g^−1^ dry soil) for bacteria, actinobacteria, fungi; and in number of organisms gram^−1^ of dry soil for: total protozoa (flagellates, ciliates and amoeba) and nematodes. This data provided microbial biomass data to determine a structural fungal and bacterial biomass ratio (F:B) and a predator/prey assessment of the different trophic levels. Sample preparation and biomass quantification implemented direct observation microscopy (visible light, differential interference contrast) and other methodologies (Ingham, 1995; Ekelund, 1998; Stamatiadis, Doran & Ingham, 1990).

A soil-food web proxy, for each of the ranch pairs, was assessed as a composite summation of all normalized soil food web measurements for each sampled ranch catena, offering an internal, normalized full soil food web metric to compare aggregate soil food web population dynamics between ranches.

### Soil CO_2_ respiration rates

Soil-respiration C (g C m^−2^ day^−1^) was measured with *in-situ* static alkali reactors (Gupta & Singh, 1981), using a 50 mL plastic centrifuge tube, containing 15 mL of standardized 1 M KOH, with a cross-sectional area of about 25% of soil surface sampled. Diurnal soil respiration measurements were conducted for the 24-hour period prior to, and at the identical location that soil samples were taken to assess SOC and the soil food web. Reactors were covered with a ∼1-liter glass cover (canning jar) screwed into the soil profile approximately two cm deep. Prior to placing the jar over the 50 ml centrifuge tube, all vegetation was removed from the surface for accurate evaluation of CO_2_ respired from the soil. Reactors measured soil CO_2_ emissions, over a diurnal time period, remaining in the field for 24 h (starting times and ending times were recorded to assess total time to 1-minute accuracy). After the 24-hour period, the 50 ml tubes were then removed from the reactors, capped, and taken to a laboratory for quantification of CO_2_ absorption by the 1 M KOH solution. A non-destructive analysis technique was implemented assessing reduction in reactor KOH solution conductivity as correlated to the quantity of CO_2_ absorbed, as described in Methodology S1. Respiration results were expressed in g C m^−2^ day^−1^. A small number of static-alkali reactors were disturbed by grazing livestock resulting in a different quantity of “n” comparisons.

The static alkali reactor methodology was chosen to insure identical atmospheric conditions (temperature, solar insolation, *etc*.) and field moisture conditions for simultaneous side-by-side same-day comparison of all 12 sampling locations for each AMP and CG farm.

### Statistical analyses

Univariate statistical analyses were used to test differences in means between paired and pooled measurements of AMP and CG treatments for: soil organic carbon percent (SOC%), standing crop biomass (SCB) (g dry biomass m^−2^), soil food web (µg g^−1^ dry soil and microorganism counts g^−1^ dry soil), and soil-respiration C (g C m^−2^ day^−1^), in the five ranch pairs. Statistical data analysis was conducted with Statistics Kingdom 2017 (https://www.statskingdom.com/). All data components were initially examined for normal distribution with Shapiro–Wilk (if n < 50) and/or Shapiro-Francia, and Anderson-Darling (if n > 50); *F*-tests were used to determine data variance. Means testing methodologies were selected based on the equality of variances and normality of data. Pairwise means comparisons were conducted with Two sample *t*-test (Welch’s *T*-test) if data had a normal distribution and equal variances and a Mann Whitney *U* test (Wilcoxon rank-sum) means tests was implemented if data was not distributed normally, had unequal variances or outliers. Simple linear regression analyses were conducted to test the fit of a linear model to the data. Means results are reported as mean values,  ± standard error (mean ± SE), and a significance value *α* ≤ 0.05 threshold was used to determine statistical significance. Soil food web proxy measurements involved multiple data types (µg microbial biomass g^−1^ dry soil) and entity data counts (number of microbes g^−1^ dry soil) requiring normalization to permit data to be interrogated as a soil food web proxy, using a “universal data sub-language” (Codd, 1970).

## Results

### Ranch pair selection and antecedent precipitation

The grazing-methodology adoption period in these ranch pairs ranged from 10 to 29 years in the AMP ranches (average 17.6 years) and from 6 years to 40 years in the CG ranches (average 25.6 years) providing no significant difference in years of management between AMP and CG ranches (Two sample *t*-test; T = −1.052996; DF = 6.3342; *p* = 0.3308; Effect size = 0.67) (Table 1). Total 2018 annual net primary productivity (g dry biomass m^−2^) and recovery goal (days) demonstrated no significant difference (Two sample *t*-test; *T* = 0.9018; DF = 6.7518; *p* = 0.3981; Effect size = 0.57) (Two sample Mann–Whitney; Z = −0.1054; no DF in MW; *p* = 0.9161; Effect size = 0.033) respectively (Table 1). A statistical analysis of the grazing management practices indicated significant differences between AMP vs. CG grazing management in: Average Animal Units (AU acre^−1^) (Two sample Mann–Whitney; *Z* = 2.5377; no DF in MW; *p* = 0.011; Effect size 0.8); Average Paddock Size (ha), (Two-sample *t*-test; T = −10.8336; DF = 4.3721; *p* = 0.00025; Effect size = 6.85); Average Number of Paddocks per Herd, (Two sample Mann–Whitney; *Z* = 2.0953; no DF in MW; *p* = 0.036; Effect size = 0.66); Graze Period Goal (days), (Two sample Mann–Whitney; Z = −2.5536; no DF in MW; *p* = 0.01066; Effect size = 0.81) and Rest vs. Graze Period (ratio), (Two sample Mann–Whitney; *Z* = 23.5143; no DF in MW; *p* = 0.01193; Effect size = 0.8) (Table 1), supporting the validity of the AMP vs. CG ranch-pair management selection criteria in this research project.

Precipitation maps, of the AMP/CG ranch locations (Fig. S1), available at https://water.weather.gov/precip/, indicated similar antecedent precipitation prior to the May-June sampling event, reducing the potential impact of differing rainfall amounts on ranching system SCB production. Monthly Percent-of-Normal rainfall, for January–June of 2018 and Departure-from-Normal rainfall, for April to May 2018 precipitation maps (Fig. S1) indicate that precipitation amounts supplied the historical monthly average rainfall or better at each sampling site, to ensure comparable precipitation quantities were available at each research site to support equivalent growth potential of SCB on ranch pairs.

Data from one-meter core samples, taken at the CG, and AMP area locations, on the same day by other researchers on this project, concurrent with the timing and location of microbiological sampling, supported sufficient soil water content for accurate soil carbon respiration and the soil bulk density assessment. The analysis of the core samples relayed the average soil moisture content on the AMP, and CG sites was ∼17.56% ± 1.1%. Soil bulk density averaged 1.21 g cm^−3^ ± 0.03 g cm^−3^ in the top ten centimeters of both AMP and CG soil profiles (Mosier et al., 2021).

### Soil organic carbon

Soil organic carbon percentages, SOC (%), from AMP management strategies were significantly greater when compared to CG ranches in three of the five pairwise comparisons: AMP-3/CG-3, (Two sample *t*-test; *T* = 6.3614; DF = 20.99; *p* = 2.62 ×10^−6^; Effect size = 2.6); AMP-4/CG-4, (Two sample Mann–Whitney; *Z* = 2.3393; no DF in MW; *p* = 0.0193; Effect size= 0.48); and AMP-5/CG-5, (Two sample *t*-test; *T* = 6.1855; DF = 21.5438 ; *p* = 3.474 ×10^−6^; Effect size = 2.52) (Table 2). There was no significant difference of SOC (%) in AMP-1/CG-1 and AMP-2/CG-2.

**Table 2:** Adaptive multi-paddock and continuous grazing standing crop biomass, soil organic carbon and soil respiration. AMP and CG pairwise and pooled comparison of standing crop biomass (g dry biomass m^−2^); soil organic carbon (%); and soil respiration (g C m^−2^ day^−1^). Statistically significant comparisons are highlighted in bold type.

Pairwise comparison		**Standing crop biomass (g dry biomass 0.1 m^2^)**		**Soil organic carbon (%)**		**Respiration (g C m^−2^ day^−1^)**	
	**AMP-1**	241.7 ± 18.6	NS	2.59 ± 0.11	NS	2.477 ± 0.151	NS
	**CG-1**	212.1 ± 15.1		2.97 ± 0.17		2.798 ± 0.302	
	**AMP-2**	216.9 ± 24.0	*p* = .0011	2.44 ± 0.12	NS	**N/A**
	**CG-2**	**362.3** ± **31.2**		2.35 ± 0.10		
	**AMP-3**	**380.6** ± **24.2**	*p* = 0.0011	**2.28** ± **0.10**	*p* = 2.62×10^−6^	1.408 ± 0.210	*p* = 0.017
	**CG-3**	95.5 ± 6.0		1.49 ± 0.08		**2.135** ± **0.141**	
	**AMP-4**	**293.2** ± **29.0**	*p* = 2.1× 10^−6^	**2.39** ± **0.17**	*p* = 0.0193	2.623 ± 0.223	NS
	**CG-4**	122.0 ± 5.9		1.87 ± 0.08		1.855 ± 0.281	
	**AMP-5**	**350.0** ± **20.8**	***p* = 2.007 × 10** ^−6^	**4.49** ± **0.17**	***p* = 3.47 × 10** ^−6^	2.453 ± 0.197	***p* = 0.0023**
	**CG-5**	215.5 ± 14.1		3.08 ± 0.15		**3.889** ± **0.323**	
**Pooled comparison**		**Standing crop biomass (g dry biomass m^2^)**		**Soil organic carbon (%)**		**Respiration** **(g C m^−2^ day^−1^)**	
	**AMP**	**294.14** ± **11.4326**	***p* = 6.26 × 10** ^−10^	**2.83817** ± **0.12328**	***p* = 0.008**	2.2372 ± 0.1312	***p* = 0.0221**
	**CG**	201.45 ± 10.774		2.35418 ± 0.09524		**2.7799** ± **0.1891**	

**Notes.**

Abbreviations NS No Statistical Significance N/A data not available

Pooled AMP soil organic carbon exhibited mean cumulative ranch SOC (%) of 2.83 ± 0.123%C, or 21% greater than mean cumulative CG SOC (%) of 2.354 ± 0.09%C (Two sample Mann–Whitney; *Z* = 2.6507; no DF in MW; *p* = 0.008; Effect size = 0.24) (Table 2).

### Standing crop biomass (SCB)

Pairwise comparisons of standing crop biomass (SCB) were significantly greater in AMP systems in three of the five pairwise comparisons AMP-3/CG-3, (Two sample *t*-test Mann–Whitney; Z = −3.2627; no DF in MW; *p* = 0.0011; Effect size = 0.42); AMP-4/CG-4, (Two sample *t*-test; DF = 31.4136; T = −5.7858p = 2.1 ×10^−6^; Effect size = 1.49) and AMP-5/CG-5, (Two sample *t*-test; *T* = 5.31036; DF = 51.0642; *p* = 2.007 ×10^−6^; Effect size = 1.38) (Table 2); and reversed in the AMP-2/CG-2 comparison (Two sample Mann–Whitney; Z = −3.2627; no DF in MW; *p* = 0.0011; Effect size = 0.42) and not statistically significant in the AMP-1/CG-1 ranch pair.

AMP management strategies exhibited a pooled SCB mean of 294.1  ± 11.43 g dry biomass m^−2^, ∼92.7 g dry biomass or ∼46% more SCB than pooled CG systems SCB mean of 201.5  ± 10.74 g dry biomass m^−2^ (Two sample Mann–Whitney; *Z* = 6.1836; no DF in MW; *p* = 6.26 ×10^−10^; Effect size = 0.35) (Table 2).

### Soil food web

Pairwise comparisons of AMP and CG soil food web analyses provided only one farm pair, AMP-3/CG-3, that demonstrated statistically significant differences in the means of soil food web- bacteria, (Two sample *t*-test; T = −2.8435; DF = 16.003; *p* = 0.0117; Effect size = 1.16); F:B ratio, (Two sample Mann–Whitney; *Z* = 2.1096; no DF in MW; *p* = 0.03489; Effect size = 0.43); soil food web proxy, (Two sample Mann–Whitney; *Z* = 2.5094; no DF in MW; *p* = 0.01209; Effect size = 0.5); and protozoa enumeration, (Two sample Mann–Whitney; *Z* = 2.4858; no DF in MW: *p* = 0.01292; Effect size = 0.51) (Table S2).

Pooled comparison of AMP and CG systems indicated a significant difference in the means of the bacterial population (Two sample Mann–Whitney; Z = −2.1766; no Df in MW; *p* = 0.02951; Effect size = 0.2) and protozoa enumeration (Two sample Mann–Whitney; *Z* = 2.3522; no DF in MW; *p* = 0.01866; Effect size = 0.21) (Table S2) but no statistically significant differences were observed in fungi, F:B ratio or soil foodweb proxy.

**Figure 1:**
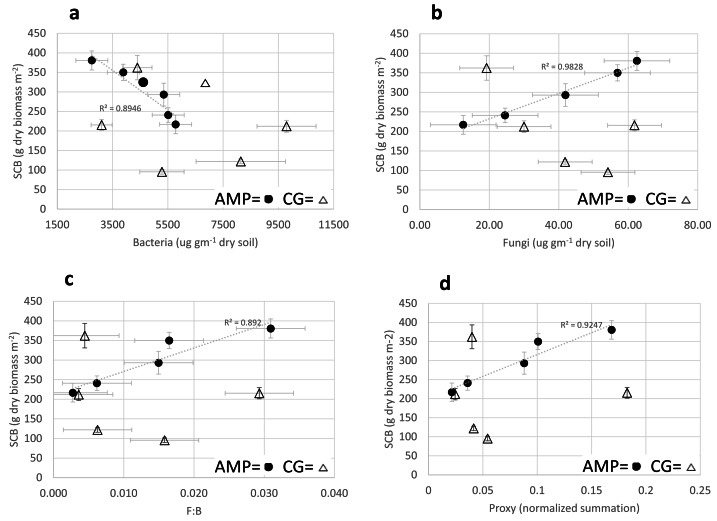
Comparison of adaptive multi-paddock and continuous grazing soil food web. Comparison of Adaptive Multi-Paddock (AMP) and Continuous Grazing (CG) standing crop biomass (SCB) (g dry biomass m^−2^) with bacterial and fungal biomass (A and B) (ug g^−1^ dry soil) and F:B ratio (C) and a Foodweb Proxy (D) (a normalized assessment of the soil foodweb). AMP icons are black filled circles, CG icons are open triangles.

Simple linear regression analyses of mean AMP soil food web variables with SCB provided very strong direct relationships with fungal biomass, (*R* = 0.9915; F(1,3) = 175.35; *p* = 0.015); F:B ratio (*R* = 0.9371; F(1,3) = 21.56; *p* = 0.019); and soil food web proxy (*R* = 0.9616; F(1,3) = 36.75; *p* = 0.009) (Figs. 1B–1D), and a very strong inverse relationship with bacterial biomass (R = −0.946; F(1,3) = 25.56; *p* = 0.015) (Fig. 1A).

Simple linear regression analyses of CG soil food web variables with SCB for bacteria biomass, fungal biomass, F:B ratio and soil food web proxy were not statistically significant; (R = −0.3061, F(1,3) = 0.31, *p* = 0.6165); (R = −0.6605, F(1,3) = 2.32, *p* = 0.225); (R = −0. 2386; F(1,3) = 0.18, *p* = 0.6691); (*R* = 0.02491, F(1,3) = 0.00016, *p* = .991) respectively (Figs. 1B–1D) (trendlines not shown).

**Figure 2:**
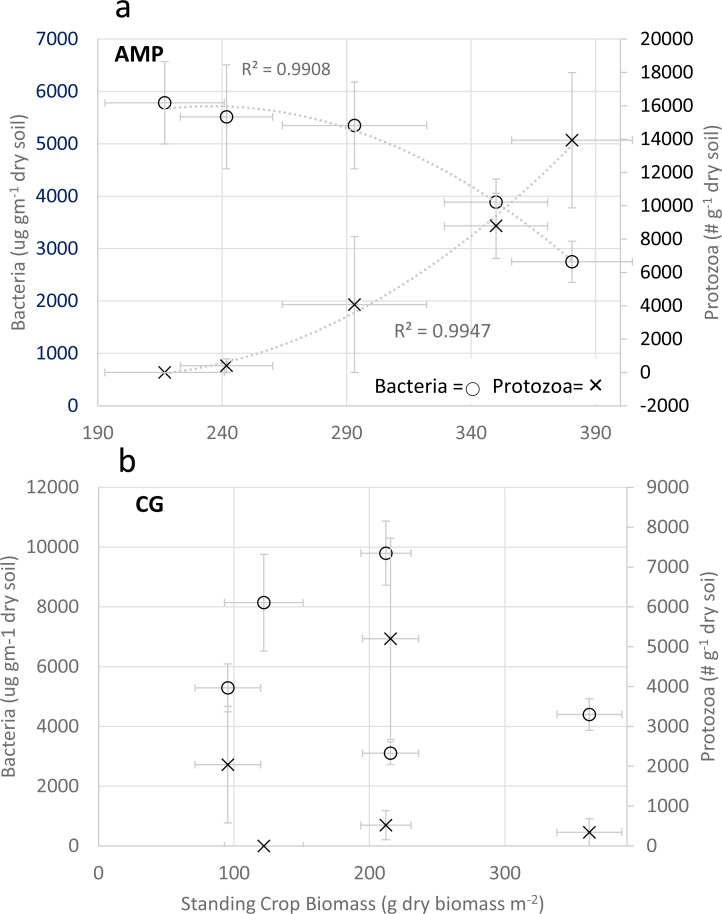
Comparison of Predator/Prey relationships for bacteria and protozoa. Comparison of Adaptive Multi-Paddock (AMP) and Continuous Grazing (CG) bacterial biomass (ug g^−1^ dry soil), protozoa enumeration (# g^−1^ dry soil); and standing crop biomass (g dry biomass m^−2^). (A) Comparison of these three variables in AMP systems and (B) comparison of these three variables in CG systems. Bacteria = open circles Protozoa = **x**.

Predator/prey relationships, comparing protozoa populations (# g^−1^ dry soil) and bacteria biomass (µg g^−1^ dry soil) with standing crop biomass in AMP systems, provided a very strong positive relationship to total protozoa (*R* = 0.9826; F(1,3) = 83.68; *p* = 0.003), and a strong inverse relationship to bacterial biomass (R = −0.946; F(1,3) = 25.56; *p* = 0.015) (Fig. 2A); where CG systems provided no significant comparisons (Fig. 2B).

### Soil respiration

Pairwise comparisons of AMP and CG soil respiration provided two farm pairs, AMP-3/CG-3 (Two sample *t*-test; T = −2.8734; DF = 9.4854; *p* = 0.01744; Effect size = 1.54) and AMP-5, CG-5 (Two sample *t*-test; T = −3.797; DF = 12.7065; *p* = 0.0023; Effect size = 1.78) that demonstrated statistically significant differences in respiration (Table 2).

Pooled AMP management soil carbon respiration exhibited 19.52% less mean cumulative soil respiration (2.237 ± 0.131 g C m^−2^ day^−1^) when compared to CG soil respiration (2.779 ± 0.1890 g C m^−2^ day^−1^) (Two sample *t*-test; T = −2.3581; DF = 52.3541; *p* = 0.0221; Effect size = 0.59) (Table 2). Carbon dioxide flux towers have been installed by associated researchers on this project and are currently tracking soil respiration on two of the farm pairs AMP-4/CG-4 and AMP-5/CG-5. Data is still being collected but their initial data is also observing a commensurate ∼20% reduction in soil C (CO_2_) respiration comparing AMP to CG management (R. Clement, 2022, Pers. Comm.).

## Discussion

Documentation of the influence that AMP and CG management systems have on soil food web population and structure in grazing lands is scarce. This research focused on evaluating AMP and CG grazing management’s impact on soil food web population and structure and their effect on, (1) system standing crop biomass production or photosynthetic capacity, (2) SOC carbon accrual and (3) soil CO_2_ respiration on grazing lands.

Detailed understanding of the soil food web relationship to ecosystem function has often proven to be complicated, as have the development of methods to accurately assess soil food web structure. Much of this difficulty is due to our inability to make accurate direct observations, such as the technical challenges of measuring *in situ* activities, and the high diversity and/or spatial heterogeneity of the soil food web and soil environment (Barns, Takala & Kuske, 1999; Torsvik, Ovreas & Thingstad, 2002; Strickland & Rousk, 2010; Malik et al., 2016; Johnson, 2017).

This point-in-time research approach attempts to address the many challenges long-term research projects encounter *i.e.*, multi-year, multiple sampling events, unpredictable weather conditions, participant buy-in, commitment and financial stability, adherence to prescribed practices, and duration of experiment. In addition to these challenges are the logistics of finding multiple adjacent ranches that have been practicing AMP and CG grazing for a minimum of one decade along with the significant financial investment and multi-year commitment by researchers, funders and land-owners. A point-in-time research approach can be a timely and cost-effective approach to garner the necessary data to determine the impact and/or efficacy of AMP and CG management practices on rangeland health and rancher productivity. Point-in-time research may also offer preliminary data or logistical design components that may benefit planning of long-term research opportunities. If ranchers and policymakers need critical data to influence their decisions towards adoption of AMP or CG management, then a point-in-time research project may be the best path forward to achieve those agendas in a timely manner.

Standing crop biomass is a keystone metric for determining soil fertility and productivity of AMP and CG management systems. Pairwise comparisons of AMP and CG farm pairs for the individual measurements of SCB, SOC (%), multiple soil food web components and soil respiration revealed very few clues for differentiating management practices, as results fluctuated and varied as related to the paired research farms (Table 2, Table S2). However; when the soil food web components were placed in the context of standing crop biomass productivity, simple linear regression analyses of the variables measured in pooled comparisons of AMP and CG management provided significant insight into the impact that soil food web structures have in AMP grazing for improving SCB, promoting SOC (%) increases and reducing soil C (CO_2_) respiration.

Extensive sampling (*n* = 12) of the soil food web components, at each participating AMP and CG farm, helped buffer the inherent heterogeneity of the soil food web matrix and provide valid comparisons with which to assess the influence of each soil food web component singularly and collectively. Direct pairwise comparisons of soil food web data between AMP and CG managed grazing offered little information for defining differences in grazing management systems, except in the AMP-3/CG-3 ranch pair. Soil food web components, bacteria, F:B ratio, soil food web proxy and total protozoa all demonstrated statistically significant differences between AMP and CG management in the AMP-3/CG-3 ranch pair. Of all the AMP/CG ranch pairs the AMP-3/CG-3 ranch comparison demonstrated the most dramatic contrast of management outcomes for pasture health and productivity when applying visual field observations, The AMP-3 ranch had the highest SCB and the lowest soil respiration of all ranches and it has the potential to provide a view to the future for the benefits that could be achieved as it had the longest period of the adoption of AMP practices when compared to all other AMP systems.

Comparisons of AMP and CG soil food web variables to standing crop biomass, were the most informative as they revealed very strong positive linear correlations of AMP systems increase in SCB to fungal biomass, F:B ratio, and a soil food web proxy while demonstrating very strong inverse relationships to bacterial biomass (Fig. 1). AMP-grazing measured variables correlated extremely well with SCB but CG systems demonstrated a complete lack of functional correlation (Fig. 1). The application of the soil food web analyses, as a potentially reliable metric of soil health in AMP farms, demonstrated good potential to resolve soil health and be predictive of SCB; however, no similar relationships were evident on CG farms. This may help explain the observations, by some researchers, relying on soil food web or soil fungal-to-bacterial ratios (F:B) to be predictive of: (a) the turnover of soil organic matter (Rousk & Frey, 2015), (b) growth of vegetation (Wong et al., 2015), or (c) enhancement of carbon storage in soils that have a more fungal dominant soil food web (Thiet, Freya & Six, 2006).

The question arises why do the soil microbiomes in AMP systems demonstrate strong correlations with SCB, and offer little statistically significant correlations in CG systems? There are likely several potential mechanisms, working singularly or collectively, that might help explain why the soil food web components in AMP systems correlated so well with SCB. Primary of these would be energy influx (de Vries et al., 2013), followed by nutrient availability, (Estrela et al., 2022), plant litter deposition (Heijboer et al., 2018), succession of microbial groups during the decomposition of litter (Keeley, 1987), nutrient deficiency-initiated dormancy (Cohen, 1966), the application of synthetic fertilizers, herbicides and pesticides (Birkhofer et al., 2008), and the development of predator/prey relationships in the soil food web (Brose et al., 2019).

Energy flow and directionality through a soil ecosystem is determined by the structure of the soil food web and its community of microorganisms (de Vries et al., 2013). The microbiome in soil systems represent multiple species of microorganisms operating as a complex self-organizing system demonstrating a certain level of species stability promoting processes involving conversion of energy substrates and conservation (Verstraete, 2015). Soil bacteria and fungi self-assemble into dynamic co-evolving communities, promoting complex interactions, ranging from antagonism to mutualisms, to compete or cooperate for the acquisition of nutrients driving ecosystem functions that are important for plant and animal health (Deveau et al., 2018).

Research indicates that nutrient availability governs microbial community level function and taxonomic structure (Estrela et al., 2022). Energy influx into a regime that has limited resources promotes a “unidirectional flow of energy” that is converted through the consumption and production of secondary-metabolites in an orderly succession promoting microbiome structures that utilize energy resources in a linear succession with each microorganism in that sequence having limited nutrient utilization capabilities (Marsland III et al., 2019). In contrast, a regime with a higher energy influx develops a qualitatively different structure providing simultaneous energy influxes into multiple entry points and feedback loops where community members utilize multiple energy resources with the incoming flux spread evenly over a community offering a more diverse and stable microbiome (Marsland III et al., 2019).

AMP systems demonstrated 46% more energy influx as an increase in forage productivity, when compared to CG systems, providing more total energy resource input for the grazing system. AMP systems also promoted the increase in available nutrients and energy by leaving 60% of standing crop biomass in the field as either trampled biomass (plant litter) or photosynthetically active plant material. CG systems management protocols offer limited and periodic access of standing crop biomass as a nutrient and energy substrate for the soil microbiome due to grazing protocols that remove grass down to the soil surface, requiring more time to rebuild plant structure and photosynthetic capacity while also negating the buildup of any significant soil-surface plant residue to supply energy and nutrients to support soil microbiome maintenance and function.

Research indicates that a succession of microbial groups occurs during the decomposition of litter. It was observed that ^13^C-labeled litter is first partitioned into fungal biomass with follow up partitioning into gram-negative bacteria, gram-positive bacteria, actinomycetes and micro-fauna (Heijboer et al., 2018). This successional partitioning of ^13^C into fungal biomass could help explain the differences observed between the soil microbial community structure and its correlation to SCB in AMP and not in CG grazing.

Another potential component that could affect AMP and CG soil foodweb structures is dormancy. Random or variable nutrient resource availability can determine when organisms enter or exit periods of reduced metabolic activity or dormancy (Keeley, 1987). AMP grazing protocols provide a more consistent nutrient resource by leaving ∼60% standing crop biomass in the field to feed the resident soil microbiomes where CG systems are more prone to a lack of nutrient resource availability, for the soil food web, due to removal of large percentages of plant biomass and little soil-surface residue buildup. Dormancy is only beneficial if the dormant organism does not deplete its energy reserves or avoids predation during the period that organism is dormant (Cohen, 1966). If there is a survival issue then dormancy could be another driver that promoted changes in soil food web structure and the lack of correlation of CG system’s soil food web structure with SCB.

Soil function has also been observed to diminish from the application of inorganic fertilizers, herbicides, pesticides, and parasiticides (Bardgett & McAlister, 1999; Leake et al., 2004; de Vries et al., 2006; Birkhofer et al., 2008; LaCanne & Lundgren, 2018) and application of these amendments is prominent in the CG managed ranches. Any or all of these interactions, listed above, acting singularly, collectively or interactively, could help explain the dysbiosis of the soil food web in CG managed systems but determination of which component(s) are responsible is beyond the scope of this research design.

It has been observed that high predator–prey (mass) may help stabilize communities and maintain ecosystem function (Brose, Archambault & Barnes, 2019). Pooled comparisons of bacterial biomass and protozoa enumeration with SCB revealed the importance of predator/prey interactions for their potential to liberate nutrients to facilitate plant growth (Fig. 2). The potential for improved nutrient cycling to increase SCB was very predictive in AMP systems when comparing bacterial biomass (µg gm^−1^ dry soil) and total protozoan counts (number gm^−1^ dry soil) with SCB productivity (Fig. 2A). There were very strong correlations of increases in SCB productivity as bacterial biomass decreased and total protozoan numbers increased, potentially indicating protozoa feeding on the bacterial community was promoting nutrient cycling. This predator/prey relationship was active in AMP management systems (Fig. 2A) but appeared disassociated when compared to CG management systems (Fig. 2B). Bacterial populations in AMP systems were less than 6,000 µg gm^−1^ dry soil where CG systems ranged as high as 10,000 µg gm^−1^ dry soil. Total protozoan counts exceeded 14,000 protozoa gm^−1^ dry soil in AMP systems and never exceeded 5,500 protozoa gm^−1^ dry soil in CG systems. CG systems demonstrated no significant predator/prey correlations when comparing bacteria biomass and total protozoan counts to SCB (Fig. 2B).

There is some indication that AMP systems continue to improve productivity the longer these practices are implemented. A comparison of the years of adoption with standing crop biomass for all five of the AMP grazers provides a moderate direct correlation (*R* = 0.5274; F(1,3) = 1.16; *p* = 0.361) (Fig. S2A), but when the AMP-5 farm is removed, a very strong direct relationship (*R* = 0.9589; F(1,2) = 22.83; *p* = 0.041) of years of adoption to standing crop biomass is observed (Fig. S2B). CG systems provided no similar observations as there is no diversion of plant biomass towards development of the soil food web population, structure, diversity and metabolic functionality. Leaving 60% of SCB in AMP managed systems promoted better conversion of incoming sunlight and water resources to enhance system photosynthetic capacity and resultant SCB, enough to provide sufficient forage biomass to support grazer stocking densities 2.38 times higher than CG systems (Table 1), while also promoting development of soil food web structure, diversity and functionality and collectively making AMP systems more ecologically resilient, sustainable and profitable.

Current rangeland research underestimates the potential for carbon capture on rangeland by presuming: (a) the “drivers of soil carbon fluxes on rangelands are dominated by climate rather than management” (Brown et al., 2010); (b) there is a general trending decrease in carbon sequestration potential associated with longevity of the grazing management practices (Derner & Schuman, 2007); and (c) estimates for soil carbon increases on rangeland of from 0.1 to 0.3 tonnes C ha^−1^ year^−1^ (Morgan et al., 2010). Comparison of AMP and CG management strategies challenges these observations by: (a) Brown et al., that climate instead of management is a key determiner of soil carbon flux on rangelands by demonstrating that grazing management is key to determining soil carbon flux as demonstrated by increased SCB, SOC% and reduced respiration across three climate zones (Zones 3,4 and 5); (b) Derner and Schuman’s research conclusions of a “trending decrease in carbon sequestration potential” associated with longevity of grazing management, where the number of years of adoption of AMP management increased carbon sequestration potential by demonstrating increasing SCB, SOC% and reduced soil respiration from increased longevity of AMP adoption, and (3) Morgan et al., of estimates of 0.1 to 0.3 tonnes C ha^−1^ yr^−1^ where AMP management demonstrated an extra 0.33 tonnes C ha^−1^ yr^−1^ (average SOC% increase of 0.48%, in the top 10 cm of the soil profile, with a soil with a bulk density of 1.21 g cm^−3^, over the average 17.6 years of adoption) when compared to CG systems. Sampling in the top ten centimeters does not allow a comprehensive assessment of soil carbon increase but an associated study, conducted in parallel and on the same calendar dates and identical sampling locations on these AMP and CG farms, concluded a total increase of 9 tonnes C ha^−1^ in soil organic C stocks, in the top meter of the soil, when comparing AMP to CG management (Mosier et al., 2021). This differential in soil carbon provides an average increase of 0.51 tonnes C ha^−1^ year^−1^ if calculated over the average 17.6 years of adoption of AMP practices.

Hypothetically, soil organic carbon increases of 0.51 tonnes C ha^−1^ yr^−1^ would provide an extra 1.86 tonnes CO_2_ ha^−1^ yr^−1^ removed from the atmosphere, or an extra 2.34 billion tonnes CO_2_ globally, if extrapolated to the adoption of AMP on the 1.25 billion hectares of rangeland. This mass is ∼6.39% of the total 36.6 tonnes anthropogenic CO_2_ emitted into the atmosphere.

Given photosynthetic capacity for savannahs average ∼900 g dry biomass m^−2^ yr^−1^ and temperate grasslands produce ∼700 g dry biomass m^−2^ yr^−1^ mean net primary productivity (Whittaker & Likens, 1975; Ruimy, Saugier & Dedieu, 1994), then AMP system’s 46.3% increase in SCB could hypothetically demonstrate from 323 g to 416 g SCB m^−2^ year^−1^ improvement in SCB. An average increase of 369.5 g dry biomass m^−2^ year^−1^of SCB in rangeland productivity would theoretically remove an extra 2.14 tonnes C ha^−1^ year^−1^ as a result of this increase in photosynthetic capacity. If AMP grazing methodologies were realized over the1.25 billion hectares of similar global grazing-land ecotypes, this increase in SCB would remove an extra 9.82 billion tonnes CO_2_ year ^−1^ or ∼26.8% of the 36.6 tonnes of total anthropogenic CO_2_ emissions.

Respiration can be variable with climate, temperature, moisture and season; however, the observed 19.52% reduction of soil CO_2_ respiration rates in AMP managed ranches, offers significant potential annual soil CO_2_ respiration reduction. Using a conservative average of 745 ± 421 g C m^−2^ year^−1^ for the temperate grassland and savannah annual respiration rate (Bond-Lamberty & Thomson, 2010) the 19.52% decrease in soil CO_2_ respiration could reduce soil CO_2_ emissions by 145.42 g C m^−2^ yr^−1^ (533.22 g CO_2_ m^−2^ yr^−1^). This would give a hypothetical global CO_2_ reduction potential of ∼6.66 billion tonnes CO_2_ year^−1^ on the 1.25 billion hectares of savannahs and grasslands, or approximately 18.19% of global CO_2_ emissions.

AMP grazing management offers beneficial ecosystem services from related increases of carbon compartmentalization into SOC, SCB and observed reduction in soil carbon respiration. These increases/reductions are not cumulative, but they offer the potential impact for improving overall grazing land’s efficiency, in AMP managed systems, to assist in capturing or retaining carbon in soils of rangeland that in aggregate represent ∼51.38% of anthropogenic CO_2_ emissions. These potential “efficiency” increases, resulting from the adoption of AMP grazing systems practices, could provide a path towards a cost effective, logical and viable carbon sequestration vehicle.

## Conclusions

The experimental hypothesis in this research was that AMP grazing methodologies promote beneficial changes in soil food web population and structural composition (bacterial and fungal biomass, F:B ratio, and protozoa enumeration) and function, and that these shifts in the soil food web population structure positively influence grazing system photosynthetic capacity, soil microbial respiration efficiency and the storage of carbon in AMP grazed rangeland soils when compared to CG systems. Funding for a multi-year “multi-systems” research project would have been prohibitively expensive. The observations and data garnered, in this point-in-time research analysis of soil food web population and structure in five ranch-pair comparisons of AMP and CG grazing strategies, offers preliminary data and experimental design components that provide an alternative perspective for viewing grazing management systems that could help in planning long-term research. Once CG ranchers are made aware of the benefits of AMP grazing management, it would be difficult for neighboring CG ranchers to commit to not altering their management practices for a long-term research project.

AMP management practices promoted: (a) increases in system photosynthetic capacity, as evidenced by higher SCB in AMP ranch systems, yielding a cumulative 92.68 g dry biomass m^−2^ increase for AMP over CG; (b) improved SOC accumulation in AMP systems, yielding an average 20.6% SOC (%) increase over CG systems; and (c) a 19.52% lower soil respiration rate in AMP ranches relative to CG ranches (Table 2) and (d) 2.36 times more animal units ha^−1^, without applying synthetic nutrient inputs.

Implementation of AMP grazing strategies, short grazing periods, leaving adequate forage residue for ground cover, providing longer rest periods for full forage recovery from grazing, and adjusting stocking levels to match available forage levels, helps to regenerate soil food web population, structure, diversity and biological functionality. AMP grazing systems provide a common-sense, economically viable mechanism to improve productivity and profitability of rangeland managers (Teague & Kreuter, 2020) while reducing green-house gasses and improving delivery of ecosystem services and socio-ecological resilience in grazing ecosystems.

## Supplemental Information

10.7717/peerj.13750/supp-1 Supplemental Information 1 Rainfall and percent of rainfall for AMP/CG ranch pairs Monthly percent-of-normal rainfall maps for January-June of 2018, and departure-from-normal maps for April-May 2018. Black stars indicate AMP and CG farm locations.

10.7717/peerj.13750/supp-2 Supplemental Information 2 Years of AMP and CG ranch grazing management Years of Adoption of Adaptive Multi-Paddock (AMP) grazing management compared to standing crop biomass. Figure (A) depicts all five AMP farms number of years of adoption and their moderate direct correlation with standing crop biomass (R2 = 0.2782; *p* = 0.361). Figure (B) relays the linear regression analysis (R2 = 0.9194; *p* = 0.04) with the removal of AMP-5 to demonstrate a strong direct relationship indicting the longer AMP practices are employed, the greater the production of standing crop biomass.

10.7717/peerj.13750/supp-3 Supplemental Information 3 Adaptive Multi-Paddock and Continuous Grazing Rainfall Monthly percent-of-normal rainfall maps for January-June of 2018, and departure-from-normal maps for April–May 2018. Stars () indicate AMP and CG farm locations.

10.7717/peerj.13750/supp-4 Supplemental Information 4 Adaptive Multi-Paddock and Continuous Grazing Soil Food Web AMP and CG pairwise and pooled comparison of bacteria and fungi (ug gm ^−1^ dry soil); fungal to bacterial ratio (F:B); soil food web proxy, a normalized summation of bacteria, fungi, and protozoa populations and protozoa (# g ^−1^ dry soil). Statistically significant comparisons are highlighted in gray backgrounds and bold type. The abbreviation NS means No Statistical Significance.

10.7717/peerj.13750/supp-5 Supplemental Information 5 Methodology for static alkali reactor assessment Analysis methodology for assessing CO _2_ absorption with Static Alkali Reactors.

10.7717/peerj.13750/supp-6 Supplemental Information 6 Soil food web, soil organic carbon, standing crop biomass and soil respiration data

## Additional Information and Declarations

### Data Availability

The following information was supplied regarding data availability: The raw data is available in the Supplementary File.
