# Supplementary figures and images for: Adaptive multi-paddock grazing management’s influence on soil food web community structure for: increasing pasture forage production, soil organic carbon, and reducing soil respiration rates in southeastern USA ranches
Source: PeerJ. 2022 Jul 19;10:e13750. doi: 10.7717/peerj.13750 (PMC9306548; doi:10.7717/peerj.13750)

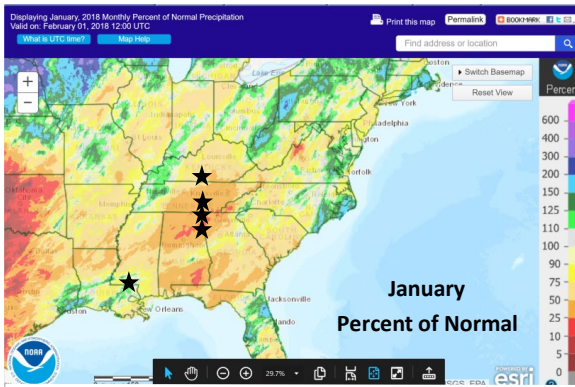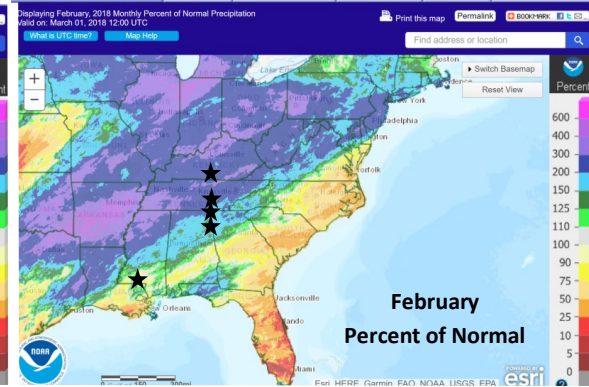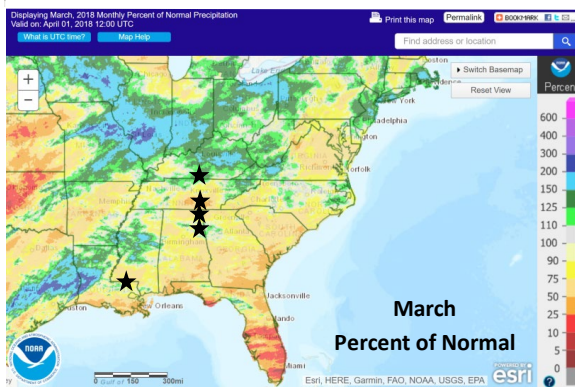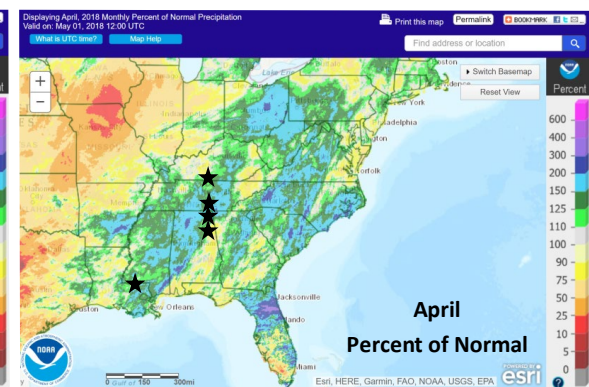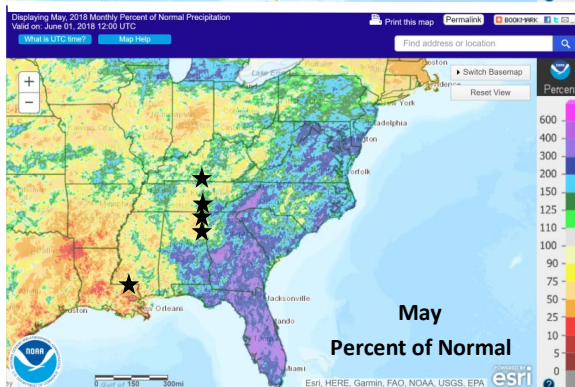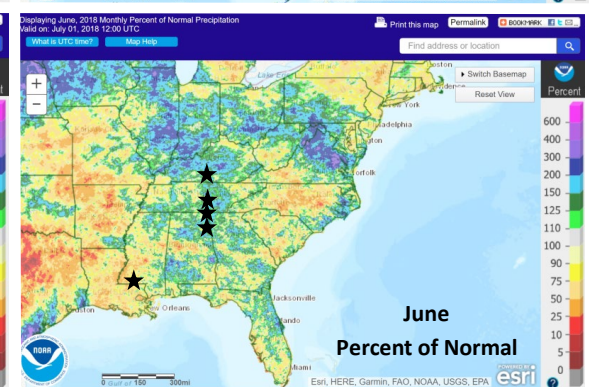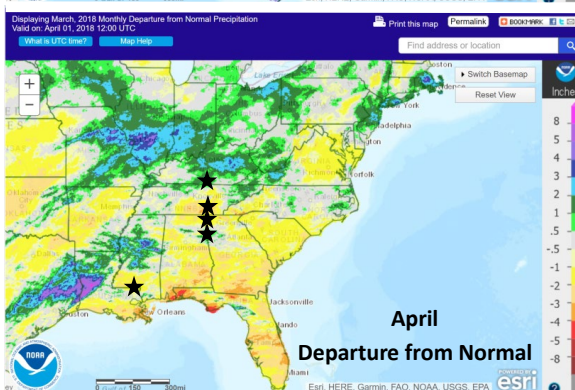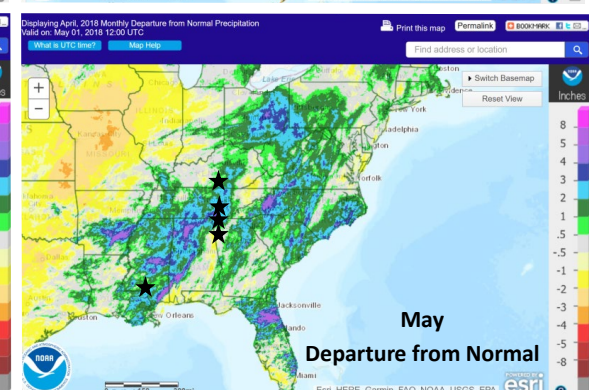

Supplement: Supplemental Information 1 — Monthly percent-of-normal rainfall maps for January-June of 2018, and departure-from-normal maps for April-May 2018. Black stars indicate AMP and CG farm locations. [file peerj-10-13750-s001.pdf]
