# Supplementary material for: Adaptive multi-paddock grazing management’s influence on soil food web community structure for: increasing pasture forage production, soil organic carbon, and reducing soil respiration rates in southeastern USA ranches
Source: PeerJ. 2022 Jul 19;10:e13750. doi: 10.7717/peerj.13750 (PMC9306548; doi:10.7717/peerj.13750)

Figure S-2- Years of Adoption of Adaptive Multi-Paddock (AMP) grazing management compared to standing crop biomass. Figure a) depicts all five AMP farms number of years of adoption and their moderate direct correlation with standing crop biomass (R^2= 0.2782; p= 0.361). Figure b) relays the linear regression analysis (R^2= 0.9194; p= 0.04) with the removal of AMP-5 to demonstrate a strong direct relationship indicting the longer AMP practices are employed, the greater the production of standing crop biomass.

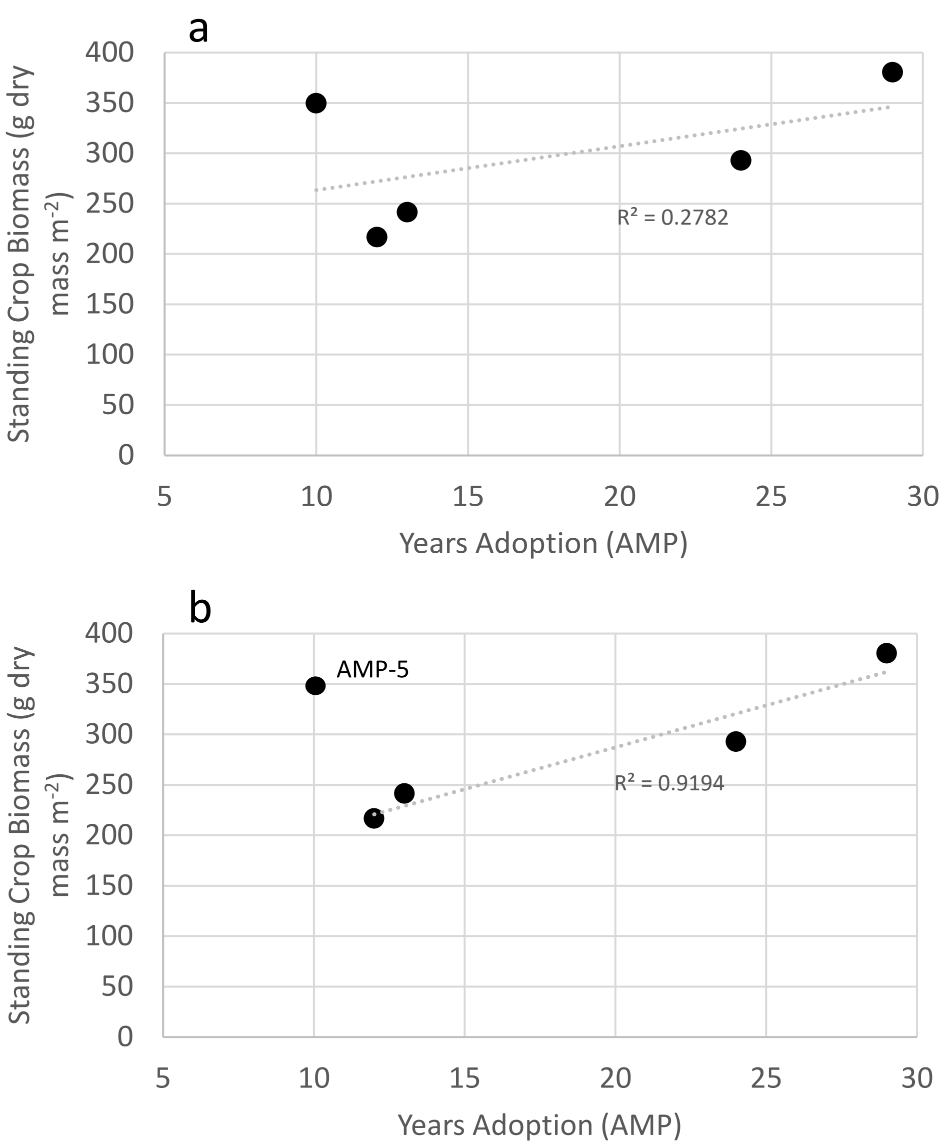

Supplement: Supplemental Information 2 — Years of Adoption of Adaptive Multi-Paddock (AMP) grazing management compared to standing crop biomass. Figure (A) depicts all five AMP farms number of years of adoption and their moderate direct correlation with standing crop biomass (R2 = 0.2782; p = 0.361). Figure (B) relays the linear regression analysis (R2 = 0.9194; p = 0.04) with the removal of AMP-5 to demonstrate a strong direct relationship indicting the longer AMP practices are employed, the greater the production of standing crop biomass. [file peerj-10-13750-s002.pdf]
