# Supplementary material for: Adaptive multi-paddock grazing management’s influence on soil food web community structure for: increasing pasture forage production, soil organic carbon, and reducing soil respiration rates in southeastern USA ranches
Source: PeerJ. 2022 Jul 19;10:e13750. doi: 10.7717/peerj.13750 (PMC9306548; doi:10.7717/peerj.13750)
Supplement: Supplemental Information 3 — Monthly percent-of-normal rainfall maps for January-June of 2018, and departure-from-normal maps for April–May 2018. Stars () indicate AMP and CG farm locations. [file peerj-10-13750-s003.pdf]

Farm pair	Catena position	Soil series	Map unit	Slope%	Taxonomic classification
Pair 1	Flat	Trimble gravelly silt loam	TrB2	2-6	Fine-loamy, siliceous, semiactive, mesic Typic Paleudults
	Slope		TrC2	6-12	
Pair 2	Flat	Emory silt loam	Ea	0-2	Fine-loamy, siliceous, semiactive, thermic Typic Paleudults
	Slope	Cumberland silty clay loam	Cm	2-5	Fine, mixed, semiactive, thermic Rhodic Paleudalfs
Pair 3	Flat	Hartsell fine sandy loam	Hc	2-6	Fine-loamy, siliceous, subactive, thermic Typic Hapludults
	Slope		Hd	6-10	
Pair 4	Flat	Cumberland gravelly loam	CoB2	2-6	Fine, kaolinitic, thermic Rhodic Paledudults
	Slope	Cumberland gravelly clay loam	CrC3	6-10	Fine, mixed, semiactive, thermic Rhodic Paleudalfs
Pair 5	Flat	Loring silt loam	12B2	2-5	Fine-silty, mixed, active, thermic Oxyaquic Fragiudalfs
	Slope		12C2	5-8	
