# Supplementary material for: Adaptive multi-paddock grazing management’s influence on soil food web community structure for: increasing pasture forage production, soil organic carbon, and reducing soil respiration rates in southeastern USA ranches
Source: PeerJ. 2022 Jul 19;10:e13750. doi: 10.7717/peerj.13750 (PMC9306548; doi:10.7717/peerj.13750)
Supplement: Supplemental Information 4 — AMP and CG pairwise and pooled comparison of bacteria and fungi (ug gm −1 dry soil); fungal to bacterial ratio (F:B); soil food web proxy, a normalized summation of bacteria, fungi, and protozoa populations and protozoa (# g −1 dry soil). Statistically significant comparisons are highlighted in gray backgrounds and bold type. The abbreviation NS means No Statistical Significance. [file peerj-10-13750-s004.pdf]

		Bacteria (ug g-1 dry soil)				Fungi (ug g-1 dry soil)				F:B Ratio				Soil Foodweb (Proxy) (Normalized)				Protozoa (# g^{-1} dry soil)			
Pairwise Comparison	**AMP-1**	5,515	±	991	NS	24.55	±	10.73	NS	*0.0062*	±	*0.003*	NS	0.0359	±	0.012	NS	410	±	410	NS
	**CG-1**	9,797	±	1,069		30.00	±	8.35		*0.0036*	±	*0.001*		0.0245	±	0.009		523	±	363	
	**AMP-2**	5,782	±	786	NS	12.50	±	3.05	NS	*0.0027*	±	*0.001*	NS	0.0215	±	0.005	**p= 6.5 x 10^{-4}**	0	±	0	NS
	**CG-2**	4,398	±	527		19.23	±	11.29		*0.0044*	±	*0.002*		**0.0400**	**±**	**0.007**		341.66	±	342	
	**AMP-3**	2,750	±	393	**p= 0.0117**	62.50	±	11.88	NS	***0.0309***	***±***	***0.009***	***p= 0.0349***	**0.1684**	**±**	**0.034**	***p= 0.01209***	***13,933***	***±***	***4056***	***p= 0.0129***
	**CG-3**	**5,288**	**±**	**801**		54.17	±	22.98		*0.0158*	±	*0.007*		0.0544	±	0.015		2,041	±	1,463	
	**AMP-4**	5,352	±	828	NS	41.92	±	14.65	NS	*0.0150*	±	*0.007*	NS	0.0880	±	0.040	NS	4,075	±	4,075	NS
	**CG-4**	8,140	±	1,617		41.83	±	10.73		*0.0072*	±	*0.002*		0.0415	±	0.010		0	±	0	
	**AMP-5**	3,888	±	442	NS	56.92	±	11.90	NS	*0.0165*	±	*0.004*	NS	0.1008	±	0.015	NS	8,792	±	1,949	NS
	**CG-5**	3,105	±	381		61.82	±	17.52		*0.0293*	±	*0.015*		0.1827	±	0.066		5,200	±	2,527	

		Bacteria (ug g-1 dry soil)				Fungi (ug g-1 dry soil)				F:B Ratio				Soil Foodweb (Proxy) (Normalized)				Protozoa (# g^{-1} dry soil)			
Pooled Comparison	**AMP**	4659.492	±	345.4	***p= 0.029***	39.2034	±	5.428	NS	0.01403	±	0.003	NS	0.08402	±	0.0128	NS	**5,575**	**±**	**1,370**	***p= 0.0187***
	**CG**	**5864.283**	**±**	**427.3**		40.7	±	6.769		0.01162	±	0.003		0.06598	±	0.0144		1,543	±	590	
