# Supplementary material for: Adaptive multi-paddock grazing management’s influence on soil food web community structure for: increasing pasture forage production, soil organic carbon, and reducing soil respiration rates in southeastern USA ranches
Source: PeerJ. 2022 Jul 19;10:e13750. doi: 10.7717/peerj.13750 (PMC9306548; doi:10.7717/peerj.13750)
Supplement: Supplemental Information 5 — Analysis methodology for assessing CO 2 absorption with Static Alkali Reactors. [file peerj-10-13750-s005.docx]

**Methodology S-3.** Analysis methodology for assessing CO_2_ absorption with Static Alkali Reactors.

Reliable methodologies, for ensuring the accurate measurement of soil CO_2_ efflux are still under debate and development (Pumpanen et al., 2004; Kuzykov, 2010); therefore, due to methodological sensitivity, practical field replication and repeatability, a static-alkali-trap methodology was chosen to measure Soil-CO_2_ Respiration. Alkali traps can yield overestimates of low CO_2_ fluxes and underestimates of high CO_2_ fluxes, but they can be reliably calibrated for intermediate ranges of CO_2_ flux (Davidson et al., 2002). Accurate soil respiration measurements can be affected when the insertion of sampling collars severs root structures, when only daytime measurements are taken (Heinemeyer et al., 2011), and when the surface area of the alkali reaction vessel is less than 6% of the soil surface area sampled (Raich and Nedelhoffer, 1989).

Parameters for the proper use of static alkali reactors, in this research, followed these methodological guidelines to ensure accurate soil respiration measurements. The static alkali reactor systems have been found to provide a reliable, internally comparable analysis of soil CO_2_ emissions, as well as soil respiration values (g C m^-2^ day^-1^) within the historically observed ranges when compared to a) different ecosystems and types of vegetation (Raich and Schlesinger, 1992), and b) the flux-tower results conducted in another component of this AMP/CG systems research.

Assessment of the absorption of CO_2_ in the static alkali reactors was conducted by measuring the conductivity of the 15 ml of KOH solution. Small amounts of the original 15 ml of KOH volume evaporated over the 24-hour sampling period. Each 50 ml centrifuge tube was returned to the original 15ml volume by adding distilled water to provide an accurate assessment of solution conductivity and soil CO_2_ emission mass. Solution conductivity and related CO_2_ absorption quantification was conducted using a HACH Sension+ 5 conductivity Meter.

A calibration curve was established prior to static-alkali-reactor analysis, to determine conductivity changes in the 15 ml of 1 *M* KOH by injecting multiple, successive 5 ml injections of pure CO_2_ through a silicone septum into a sealed 40 ml vial containing the 15 ml of 1 *M* KOH. After injection, the vial was shaken vigorously for 1 minute to assist the absorption of CO_2_ into the KOH solution. After shaking, interior pressure increase was assessed from any remaining unabsorbed CO_2_ with an inverted closed-end water column, capable of maintaining a negative atmospheric pressure, by inserting a needle into the sample vial to relieve and measure any volume of remaining unabsorbed gases. No pressure buildup was observed until a total cumulative volume of ~190 ml of CO_2_ was injected into the 15 ml of 1 *M* KOH. This quantity is far above the mass of CO_2_ observed in the 24-hour-duration static alkali reactors of this research. A calibration-curve was established (y= -1.8377x + 352.92, R^2^ = 0.9978) relative to the injected volumes of CO_2_ and implemented to assess CO_2_ mass absorbed into the 15 ml of 1 *M* KOH in the static alkali reactors used in the field to quantify soil CO_2_ respiration.
